# Establishment of high-throughput screening HTRF assay for identification small molecule inhibitors of Skp2-Cks1

**DOI:** 10.1038/s41598-021-00646-3

**Published:** 2021-10-26

**Authors:** Kaizhao Hu, Xiao-Jing Li, Moges Dessale Asmamaw, Xiao-Jing Shi, Hong-Min Liu

**Affiliations:** 1 State Key Laboratory of Esophageal Cancer Prevention and Treatment, Key Laboratory of Advanced Drug Preparation Technologies, Henan Key Laboratory of Drug Quality Control and Evaluation, Ministry of Education of China, School of Pharmaceutical Sciences, Zhengzhou University, Zhengzhou, 450001 Henan China; 2 Laboratory Animal Center, Academy of Medical Science, Zhengzhou University, Zhengzhou, 450052 Henan Province China

**Keywords:** Drug screening, Target identification, Cell-cycle proteins, Ubiquitylated proteins, Screening

## Abstract

S-phase kinase associated protein 2 (Skp2), a member of the F-box family that constitute the largest known class of ubiquitin E3 specificity components, is responsible for recognizing and recruiting cyclin-dependent kinase inhibitor p27 for its ubiquitination in the presence of the small accessory protein cyclin-dependent kinase regulatory subunit 1(Cks1). Skp2 is overexpressed in esophageal carcinoma tissues and closely related with tumor poor prognosis, and perturbation of the Skp2-Cks1 interaction by inhibitors or RNAi could inhibit the proliferation and metastasis of tumor cells. Therefore, inhibition of Skp2 function by small-molecule compounds targeting Skp2-Cks1 interaction is emerging as a promising and novel anti-cancer strategy. In this study, we establish an improved high-throughput screening platform to screen Skp2 inhibitors targeting Skp2-Cks1interaction, which may provide a new therapeutic approach for the clinic.

## Introduction

The ubiquitin–proteasome system (UPS) is responsible for much of the regulated proteolysis in the cell and has non-degradable functions as well^1^. The process of ubiquitination in vivo is a three-enzyme cascade reaction, including the activation of ubiquitin by the ubiquitin-activating enzyme(E1) followed by transfer to a ubiquitin-conjugating enzyme(E2) and linked to the target protein substrate with the ubiquitin-protein ligase (E3)^2,3^. Finally, the protein complexes, formed by E3 ubiquitin ligase and substrate via protein–protein interaction (PPI), are degraded by proteasomal complexes^4^. Therefore, protein–protein interaction is crucial in the process of ubiquitination. During tumor development, the PPI of ubiquitin–proteasome system can regulate the proliferation, metastasis, migration and drug resistance of tumor cells^5^. The PPI will be a mainstream idea of oncology drug development although there are a lot of limitations and difficulties.

**Figure 1:**
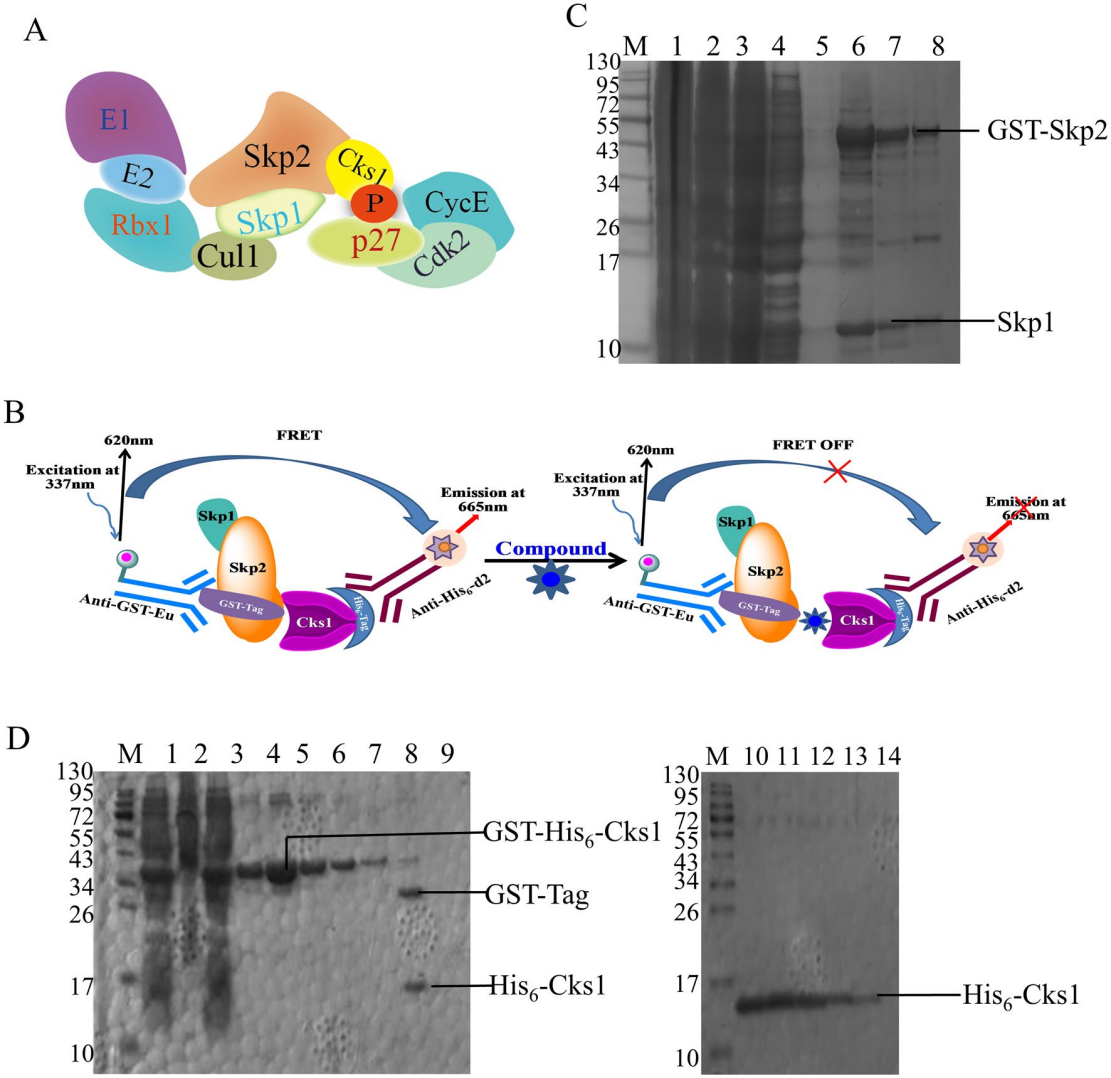
(**A**) Schematic representation of the p27-SCF-Skp2 complex. (**B**) Format of the HTRF assay to detect Skp2-Cks1 interactions. The Skp1/Skp2 complex with GST tags and Cks1 with His_6_ tags bind to the anti-GST-Eu donor beads and the anti- His_6_-d2 receptor beads, respectively. Donor and receptor beads are close to each other by GST-Skp1/Skp2 binding to His_6_-Cks1. When excited at a wavelength of 337 nm, the donor bead produces energy, one part is released at a wavelength of 620 nm, and the other part is transferred to the receptor bead by energy resonance and released at a wavelength of 665 nm. When inhibitors inhibit Skp2 binding to Cks1, only energy is released at a wavelength of 620 nm. (**C**) Purified gel and molecular sieve diagrams of protein GST-Skp1/Skp2. (**D**) Purified gel and molecular sieve diagrams of protein His_6_-Cks1. lane 9: Cut the GST-Tag by thrombin, lane 10–14: Final purification protein of His_6_-Cks1.

S-phase kinase-associated protein 2(Skp2, FBXL1 or p45), a member of the FBXL subclass of F-box proteins, is a subunit of SCF/Sk2 ubiquitin, and increased Skp2 expression is often found in cancer^6^. The discovery and development of inhibitors targeting Skp2 may promote the study of Skp2’s functions and mechanistic. Skp2 mainly degrades the Cyclin-dependent kinase inhibitor (CKIs) which can inhibit the G1 to S phase transition of the cell cycle and subsequently regulates tumor proliferation and metastasis^7^. Skp2 can induce ubiquitination of multiple substrates when it interacts with the ligand, such as p21^8^, p27^9^, p57^10^, etc. In cells, p27, a critical cell cycle regulator frequently altered in human cancer, is mainly regulated by the ubiquitin–proteasome pathway. Decreased p27 level can enhance proliferation in cancer cells and linked to poor prognosis. At the same time, the ubiquitination of p27 requires a small accessory protein, Cdc kinase subunit 1(Cks1)^11,12^ (Fig. 1A). It has been substantiated that Cks1 is essential for ubiquitin ligation of p27 in vitro reconstitution experiments^13^. The loss of Cks1 resulted in accumulation p27 in cell^14^. Inhibition of Skp2 binding to Cks1 can reduce ubiquitination degradation of p27, resulting in cycle arrest of tumor cells^15^. Therefore, the pockets of Skp2-Cks1 can be designed as a novel and credible drug target for antitumor drug development.

The methods of detecting protein–protein interaction mainly include Homogeneous time-resolved fluorescence (HTRF), AlphaScreen, TRF and Elisa^16^. The previously reported literature describing high-throughput screening assay based on HTRF for Skp2-Cks1 has some limitations^17^. The sensitivity of the high-throughput screening system decreased due to the low window value, high optimal protein concentration and steric hindrance, which may affect the screening of inhibitors.

In this study, the pocket of Skp2-Cks1 was used to construct the screening platform based on the stability of the protein and HTRF technology (Fig. 1B). We have optimized and established a high throughput screening platform for drug activity based on previous literature using purified GST-Skp2/Skp1 and His_6_-Cks1 recombinant proteins from *Escherichia coli* (DE3). The recombinant proteins GST-Skp2/Skp1 and His_6_-Cks1 bind to the anti-GST-Eu and anti-His_6_-d2, respectively. Then the fluorescence ratio of 665/620 can be detected the activity of compounds. This assay has high sensitivity and high throughput in screening the inhibitor of Skp2.

## Materials and methods

### Plasmids

The pGEX vector of GST-Skp2/Skp1^18,19^ was kindly provided by Dr. Bing Hao (University of Connecticut) and the pGEX-4T-1 vector containing glutathione S-transferase (GST)-Thrombin-His-TEV-Cks1^20,21^ was obtained from Dr. Liu Xuedong (University of Colorado).

### Expression and purification of GST-Skp2/Skp1 and His6-Cks1 proteins

The pGEX plasmid encoding GST-Skp2/Skp1 was transformed into *E. coli* BL21 (DE3) and the transformed cells were cultured in Luria–Bertani (LB) medium containing ampicillin (100 μM) and chloramphenicol (100 μM) at 37 °C with shaking until the OD_600_ reached 0.6–0.8. Then 0.5 mM isopropyl-β-d-thiogalactoside (IPTG) was added and the cultures were incubated at 16 °C overnight. On the following day the cells were collected by centrifugation at 6000 g, and lysed by ultrasonic cell disrupter (Ningbo Xinzhi Biological Technology Co., Ltd., Jiangsu, China, model: JY92-II) at 250 W in 20 mM Tris–HCl (pH = 7.5), 300 mM NaCl, 2.5 mM PMSF, and 5 mM β-mercaptoethanol. The lysate was centrifuged at 13,000 g for 10 min, and the supernatant was incubated with Glutathione (GST) resin (GE Healthcare, 17513201, USA) for 60 min at 4 °C by gently rollover. After that, the GST resin was transferred to a 30 mL Affinity Columns (Solarbio, DS0150, Beijing, China), washed by lysis buffer, and the expressed protein was eluted by elution buffer (20 mM Tris pH = 8.0, 300 mM NaCl, and 10 mM glutathione). Following GST purification, GST-Skp2/Skp1 protein was applied to a HiTrap Q HP anion exchange column (GE Healthcare, catalog number:17115301, USA) according to the manufacturer’s protocol. The column was eluted by a linear gradient of 0–1 M NaCl in 20 mM Tris–HCl buffer (pH = 7.5, 2 mM DTT) at a flow rate of 2 mL/min at 4 °C using an AKTA FPLC system (GE Healthcare, USA, model: AKTA Purifier), and the eluted fractions (usually collected for per 0.5–1 mL) were assessed via 10% SDS-PAGE (Fig. 1C). Then the eluted fractions were concentrated to 2 mL. Finally, the concentrated sample was further purified by a gel filtration column (GE Healthcare, Superdex 200 increase 10/300GL, catalog number: 28990944, USA) using an AKTA FPLC system in a buffer (pH = 7.5, 20 mM Tris–HCl, 200 mM NaCl, 2 mM DTT). The final protein solution was aliquoted, flash frozen in liquid nitrogen and stored at -80 °C. 22.62 μM GST-Skp2/Skp1 was obtained from 1 L cell culture.

The pGEX-4T-1 vector containing GST-Thrombin-His_6_-Cks1 was used to purify recombinant protein of His_6_-Cks1. After expression in *E. coli* BL21 (DE3) cells, cells were lysed and then purified by GST resin with 20 mM Tris(pH = 8.0), 300 mM NaCl and 10 mM glutathione. To remove the GST-Thrombin bp, per 1 mg of eluted GST-Thrombin -His_6_-Cks1 protein was incubated with 10 Units of thrombin overnight at 4 °C.

The SDS-PAGE was used to detect whether the GST-Thrombin bp had been removed (Fig. 1D). Then the hybrid components were concentrated to 2 mL and purified by a gel filtration column (GE Healthcare, Superdex 75 increase 10/300GL, catalog number: 29148721, USA). The final protein solution was aliquoted, flash frozen in liquid nitrogen and stored at − 80 °C. 13.87 μM His_6_-Cks1 was obtained from 1 L cell culture.

### In vitro binding assay of GST-Skp2/Skp1 with His_6_-Cks1

The pull-down assay was used to detect the in vitro binding of GST-Skp2/Skp1 with His_6_-Cks1. Briefly, GST-Skp2/Skp1 was incubated with His_6_-Cks1 by the equally molar ratio in the reaction buffer (20 mM Tris, pH = 7.5, 200 mM NaCl, 2 mM DTT) for 1 h at 4 °C. The binding reactions were then coupled to GST resin or Ni–NTA resin. The unbound proteins were washed 3 times by the reaction buffer and the bound proteins were eluted by reaction buffer containing 10 mM glutathione or 200 mM imidazole, respectively. The eluted components were separated by 10% SDS-PAGE gel and the in vitro binding of GST-Skp2/Skp1 with His6-Cks1 was identified by Coomassie brilliant blue staining.

### HTRF binding assay between GST-Skp2/Skp1 and His6-Cks1

The anti-GST antibody coupled to Eu cryptate (Cisbio Bioassays, catalog number: 61GSTKLA, Massachusetts, USA) were used for capturing GST-Skp2/Skp1 protein and anti-His6 antibody labeled with d2 donor acceptor (Cisbio Bioassays, Catalog Number: 61HISDLA, Massachusetts, USA) was chosen for combining His6-Cks1 protein. The HTRF assay was performed in OptiPlate-384 white plates (PerkinElmer, catalog number: 6007290, USA) with a total volume of 20 µL per well. GST-Skp2/Skp1-His_6_-Cks1 concentration matrix was set up these antibodies to the respective proteins in a 1:1 ratio (total antibody volume: sample volume). 5 µL GST-Skp2/Skp1 and 5 µL His_6_-Cks1 in the assay buffer (50 mM Tris pH=7.5, 0.05% BSA, 0.02% Tween-20, 150 mM NaCl, 1 mM DTT) were added to the plate and incubated in a shaker at room temperature for 20 min. Then 5 µL Mab Anti-GST-Eu cryptate beads (final conc. 0.125 µg/mL) and 5µL Mab Anti- His_6_-d2 beads mix (final conc. 1 µg/mL) in the assay buffer containing 800 mM KF were added to the mixture and incubated at room temperature for 1 h in dark. Plates were detected by microplate reader (PerkinElmer, EnVision, USA) for optimal signal detection at 620 nm and 665 nm. The ratio of 665 nm/620 nm can measure the combination of GST-Skp2/Skp1 and His_6_-Cks1.

### In vitro activity of compound based on HTRF assay of GST-Skp2/Skp1 and His_6_-Cks1

To assess the inhibitory effect of the compounds in the GST-Skp2/Skp1 and His_6_-Cks1 HTRF binding, a series of concentrations of test compounds in DMSO were pre-incubated with GST-Skp2/Skp1 protein in a shaker at room temperature for 10 min, following by conducting HTRF assay. The four-parameter Logistic regression model of GraphPad Prism software was used to calculate the IC_50_ values and inhibitory curves of the test compounds.

### Cell viability assay

The well-grown cells were seeded in 96-well plates at a density of 4 × 10^3^ cells/well. 12 h later, compounds to be tested in different concentrations were added to the plates and cultured for 72 h. Then MTT (5 mg/mL) was added with a final concentration of 0.5 mg/mL and the plates were cultured for another 4 h. The supernatant was discarded and the purple precipitate was dissolved by 150 μL of DMSO. After shaking 10 min at room temperature, the absorbance value at 490 nm wavelength was determined by a microplate reader (Tecan spark 20 M, Austria) and IC_50_ was calculated by GraphPad Prism 8.0.1 (https://www.graphpad.com/scientific-software/prism/).

### Western blot assay

Cells were seeded in a 6-well plate and treated with different concentrations of compounds for 48 h. The treated cells were harvested and lysed in RIPA buffer {25 mM Tris–HCl (pH 7.4), 150 mM NaCl, 1% NP-40, 0.1% SDS and 1% sodium deoxycholate} containing PMSF and protease inhibitor. Proteins were transferred to 0.22 μm nitrocellulose membranes via SDS-PAGE and the membranes were blocked with 5% non-fat milk in PBS for 2 h at room temperature. Then the membranes were incubated with the primary antibody in PBST (PBS buffer containing 0.05% Tween-20) overnight and the next day with secondary antibody (1:5000) in PBST for 2 h at room temperature. Finally, the signaling on the membranes was detected by enhanced chemiluminescence reagent (ECL, Thermo Scientific, USA). All antibodies were used at a 1:1000 dilution in PBST buffer containing 5% non-fat milk. The antibodies of Skp2(CST, 2652S, USA), p21(CST, 2947S, USA), p27(CST, 3686S, USA), GAPDH (CST, 5174S, USA) were obtained from Cell Signaling Technology. The full panel of western gels for Figure 4, 5 and 6 are available as Supplementary Information. 

### Cell cycle distribution assay

The cells were seeded in a 6-well plate at a density of 1.5 × 10^5^ cells/well to 80% confluency overnight, and 24 h later the cells were exposed to a specified concentration of the compounds for 48 h. Then the treated cells were collected, washed with PBS twice, and resuspended in the 75% ethanol solution overnight. Then the cells were washed with PBS for 3 times, and stained with 20 μg/mL PI (Solarbio, China) for 30 min. The final samples were detected by flow cytometry and the experimental data were processed by FlowJo v10.8 software (https://www.flowjo.com/solutions/flowjo/downloads).

### Cellular thermal shift (CETSA) assay

Cells were seeded in a cell dish overnight to 90% confluency. The cells were then treated with fresh medium containing compounds or 0.3% (v/v) DMSO at 37 °C and 5% CO_2_ for 4 h. After incubation, the cells were harvested and re-suspended in TBS. Then the cell suspension was divided evenly into 200 μL eppendorf tubes and placed in a PCR machine for carefully controlled heating to a gradient of 38–68 °C. Finally, the treated cells were lysed through repeated freeze–thaw cycles three times in liquid nitrogen-37 °C water bath, and the quantification of the target protein remaining in the soluble fraction was analyzed by western blot.

### Reverse transcription-polymerase chain reaction (RT-PCR)

After the cells were treated with compounds for 48 h, the total RNA was extracted from the cells using Ultrapure RNA Kit (Cwbio, CW0581M, China) and the RNA concentration was calculated by quantitative nucleic acid detector (Thermo Scientific, USA). After determining the purity of the isolated RNA by measuring the A260/A280 ratios, reverse transcriptase was used to reverse transcribe RNA into cDNA using the HiFiScript cDNA Synthesis Kit (Cwbio, CW2569M, China). The RT-PCR reaction volume was 20 µl containing cDNA, primers, chamQ Universal SYBR qPCR Master MIX cDNA and RNase-Free Water. The primer pairs of p27, Skp2, p21 and GAPDH (listed in Table 1) were synthesized by ShangYa gene technology company (Henan, China). Relative mRNA levels were calculated by QuantStudio Real-Time PCR Software v1.3 (https://www.thermofisher.cn/cn/zh/home/global/forms/life-science/quantstudio-6-7-flex-software.html) and normalized with the mRNA expression of GAPDH.

**Table 1:** Primer sequences of the genes for RT-PCR.

Gene name	Forward (5′–3′)	Reverse (5′–3′)
p27	GCAAGTACGAGTGGCAAGAG	CAAATGCGTGTCCTCAGAGT
Skp2	ATGCCCCAATCTTGTCCATCT	CACCGACTGAGTGATAGGTGT
p21	GAGGCAGACCAGCCTGACA	TCTGCGCTTGGAGTGATAGAAAT
GAPDH	ACAACTTTGGTATCGTGGAAGG	GCCATCACGCCACAGTTTC

### Statistical analysis

All of the data were depicted as means ± S.D. from two or three experiments. *P* values were calculated using a *t* test with *P* < 0.05 or less.

## Results

### In vitro binding between GST-Skp2/Skp1 and His_6_-Cks1

Protein–protein interaction (PPI) plays an indispensable role in regulating biological systems and is involved in the development of a variety of disease states^22^. The drug screening based on the PPI principle may be an important direction of oncology drug development, but it also faces many challenges, such as the discovery of binding pocket of protein, the stability of protein binding in vitro, the sensitivity of detection method, etc. Therefore, an appropriate method to detect the binding of two proteins is very important for the high throughput screening platform based on the principle of PPI. The sensitivity, reliability, precision, economy, and maneuverability of the method should also be considered.

**Figure 2:**
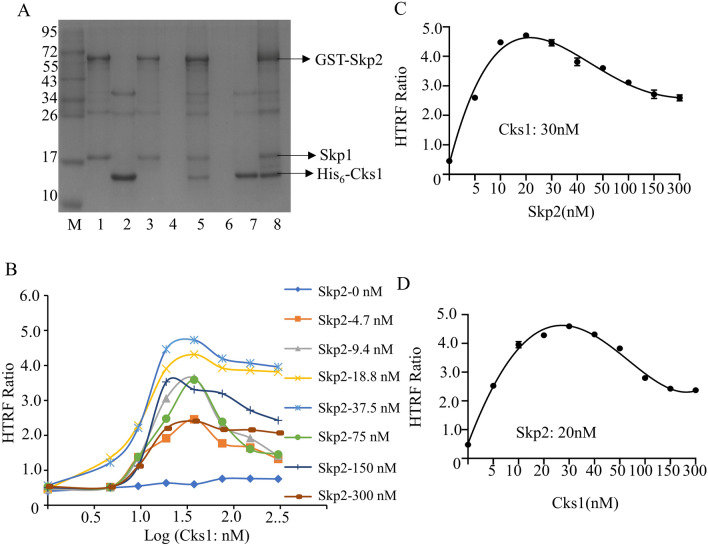
The binding experiment of GST-Skp2 and His_6_-Cks1 in vitro. (**A**) The pull-down experiment verified whether the protein was active in vitro: lane1: GST-Skp2; lane2: His_6_-Cks1; lane3: GST-Beads + GST-Skp2; lane4: GST-Beads + His_6_-Cks1; lane5: GST-Beads + GST-Skp2 + His_6_-Cks1; lane6: His-Beads + GST-Skp2; lane7: His-Beads + His_6_-Cks1; lane8: His-Beads + GST-Skp2 + His_6_-Cks1. (**B**–**D**) The protein optimal concentration of GST-Skp2 binding to His_6_-Cks1 was determined by orthogonal experiment, Skp2: 30 nM, Cks1: 20 nM.

S-Phase Kinase Associated Protein 1(Skp1) is an essential component of the SCF (Skp1-Cul1-F-box protein) ubiquitin ligase complex, which was reported to enhance the expression and stability of Skp2^23,24^. So Skp2/Skp1 is expressed with GST-tag which can firmly combine with anti-GST donor beads. At the same time, the anti-His_6_ acceptor beads can be identified with Cks1 that has the His_6_-tag. The two proteins were purified from *Escherichia coli* using the method described above. We then verified the binding ability of the two proteins in vitro through a pull-down experiment. We first tested the binding ability of GST-Skp2 and His_6_-Cks1 through GST-beads. Then reverse verify the results by His_6_-beads. The results showed that the purified protein still had good activity and binding ability in vitro (Fig. 2A).

We cross-titrated the binding of the two proteins for determining the optimum protein concentration for the HTRF assay. A series of GST-Skp2 and His_6_-Cks1 were mixed with two beads and the fluorescence signal values were measured by a microplate reader. It was shown that a higher signal of HTRF ratio has appeared when GST-Skp2 and His_6_-Cks1 were in the range of 18.75 nM to 37.5 nM (Fig. 2B). According to literature and the design of subsequent experiments, the concentration of GST-Skp2 and His_6_-Cks1 were set at 20 nM and 30 nM respectively. When the concentration of GST-Skp2was chosen to be 20 nM, it was mixed with a series of gradient concentrations of His_6_-Cks1 respectively, and the HTRF signal had the optimal value at 30 nM for His_6_-Cks1 (Fig. 2C). Similarly, when His_6_-Cks1 was set at 30 nM, GST-Skp2 had a maximum value of 20 nM (Fig. 2D).

### The optimization of HTRF assay about GST-Skp2 and His_6_-Cks1 HTS

To evaluate the stability of the Skp2/Skp1-Cks1 HTRF assay for HTS, the following parameters were tested, including the duration of HTRF signal, the concentration of DMSO and the order in which the reactants were added.

**Figure 3:**
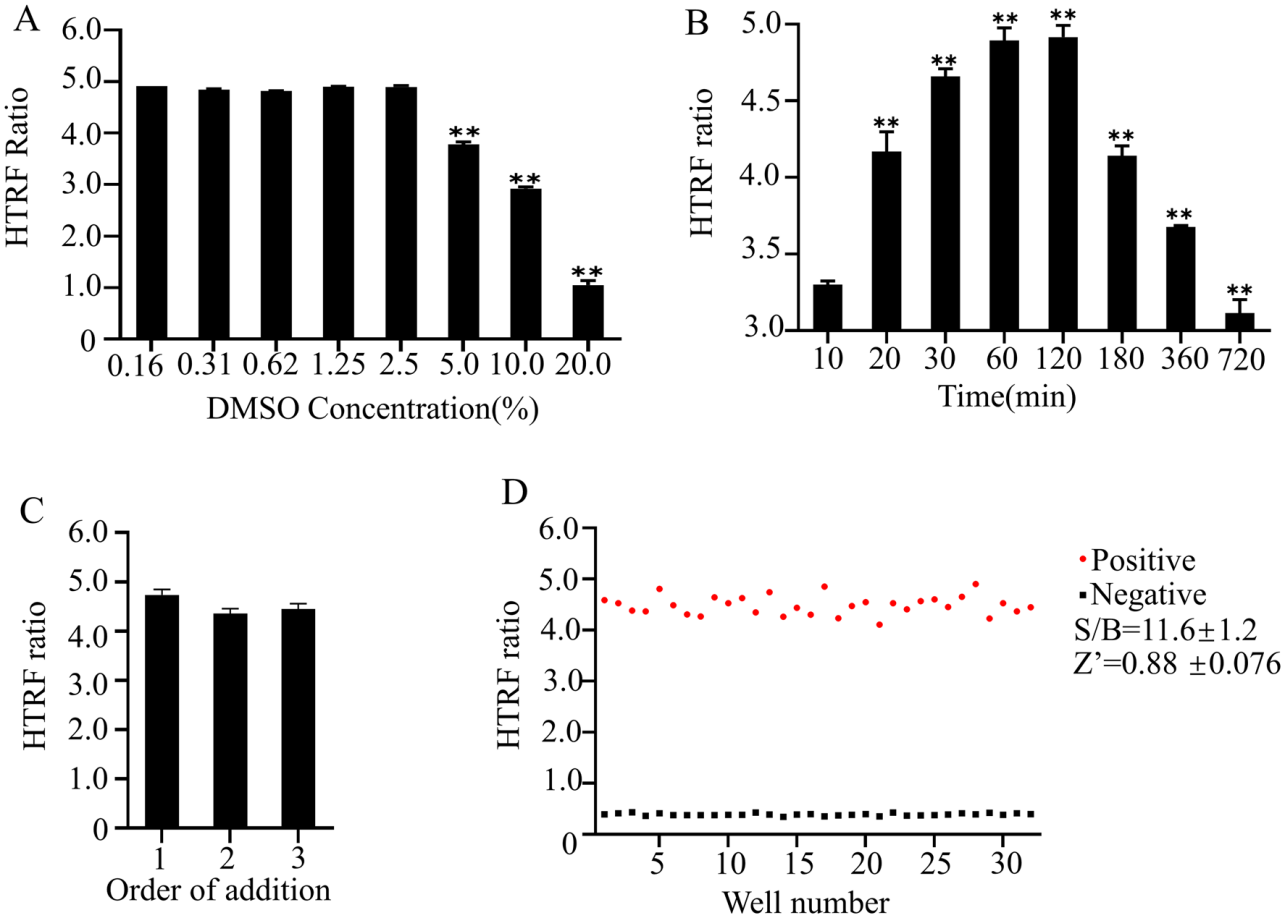
The optimization of HTRF assay about GST-Skp2 and His_6_-Cks1 HTS. (**A**) Influence of DMSO on the signal value of reaction system. (**B**) Time dependence of the HTRF signal. (**C**) Order of addition. (**D**) GST-Skp2 (20 nM) was pre-incubated with or without His_6_-Cks1(30 nM) for 60 min. S/B = 11.48, Z = 0.87. The representative data of at least three independent experiments are shown, *P < 0.05, **P < 0.01 (mean ± SD).

The screened compounds were added into the reaction system after being dissolved by DMSO. DMSO, as an organic solvent, might reduce the activity of proteins and the stability of the whole reaction system. It is necessary to determine the appropriate concentration of DMSO in the reaction system which doesn't affect the binding of Skp2 and Cks1. As was shown in the results, it had no significant effect on Skp2-Cks1 binding activity when the proportion of DMSO is within 2.5% (Fig. 3A). So, the maximum DMSO concentration we chose was 1%. The fluorescence, producing by the bind of protein and beads changes over time. Therefore, the proper incubation time was also important for a stable assay system. Next, we examined the changes in HTRF values for incubation periods up to 12 h. When Skp2 incubated for 1 h with Cks1, two antibody beads were added, and HTRF values were measured at each time point. The 384-plates should keep in dark unless the measurement time point. We found that the HTRF signal was stable between an hour and two hours (Fig. 3B). If the incubation time is less than one hour, the binding between protein and beads is not full, while the fluorescence may be quenched after two hours. It takes about 4 min to scan a 384-well plate by a microplate reader, the final incubation time was 90 min. It means that we can screen large quantities of compounds simultaneously.

Next, we investigated whether the order of addition of reagents had any effect on the interaction of the two proteins. In the present study, we tested three kinds of order about reagents added: (1) GST-Skp2 and DMSO were preincubated for 10 min, His_6_-Cks1 was added for 20 min, two mixed antibody detection reagents were added and incubated for 90 min and finally the mixture was detected by a microplate reader. (2) His_6_-Cks1 and DMSO were preincubated for 10 min, GST-Skp2 was added for 20 min, two mixed antibody detection reagents were added and incubated for 90 min and finally the mixture was detected by a microplate reader. (3) GST-Skp2 and His_6_-Cks1 were incubated with antibody detection reagent for 20 min respectively, and then mixed evenly, DMSO was added for 90 min and finally the mixture was detected by a microplate reader. We found that HTRF signal values were more stable when the compound was incubated with GST-Skp2, followed by the addition of His_6_-Cks1 and antibody detection reagent (Fig. 3C). Based on the above experimental results, a stable high-throughput screening assay could be obtained: GST-Skp2 was incubated with the compound for 10 min, His_6_-Cks1 was added for another 20 min, and the mixture was detected by a microplate reader after adding antibody detection reagent for 90 min.

To explore the stability and reliability of high-throughput screening based on the HTRF assay, repeated tests were performed at a single concentration of antibody reagents. Then the Z value and the S/B value were calculated (Z = 1—(3 *(σ_p_ + σ_n_ )∕|(μ_p_—μ_n_ ) |)). Z′ = 0.88 ± 0.076 and an average plate S/B of 11.6 ± 1.2 (Fig. 3D). This result indicated that the HTRF assay can be used to screen specific target compounds of Skp2-Cks1.

### Verification of practicability for Skp2-Cks1 inhibitors screening system

**Figure 4:**
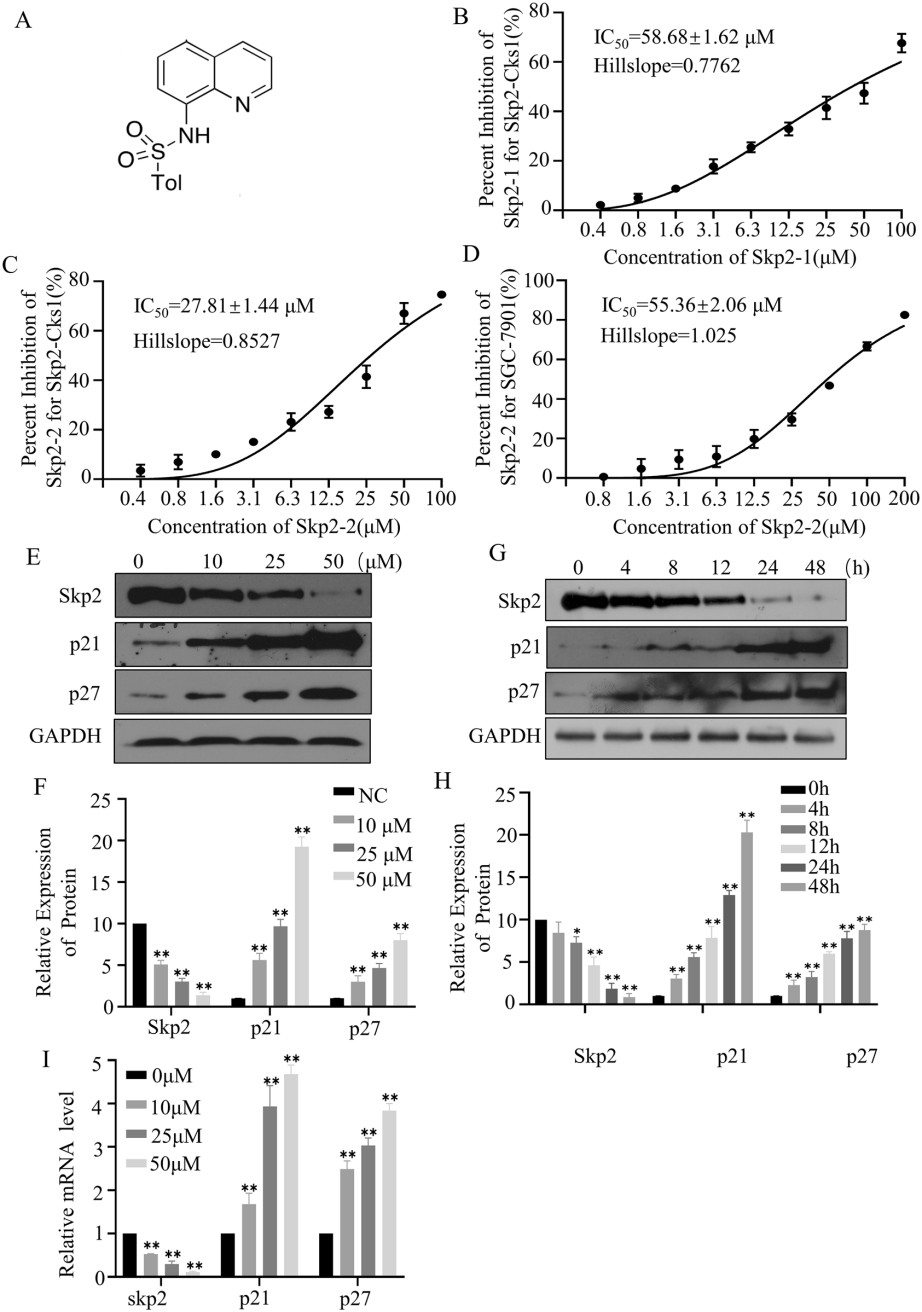
Skp2-2 inhibits biochemical pathways in SGC-7901. (**A**) The chemical structure of compound Skp2-2. (**B**,**C**) The percent inhibition of the compound Skp2-1 and Skp2-2 at various concentrations in Skp2-Cks1. (**D**) The percent inhibition of the compound Skp2-2 at various concentrations in SGC-7901. (**E**,**F**) SGC-7901 cells treated with increasing concentrations of Skp2-2 for 48 h. (**G**,**H**) SGC-7901 cells treated with 50 μM Skp2-2 for 0–48 h. The cell lysates were collected for Western blotting with the indicated antibodies. (**I**) SGC-7901cells were treated with increasing concentrations of Skp2-2 for 48 h and the changes of Skp2, p21, and p27 in mRNA level were detected by RT-PCR. The representative data of at least three independent experiments are shown, *P < 0.05, **P < 0.01. compared with the control group (mean ± SD, n = 3).

To verify the usefulness of this screening system, we used compounds Skp2-1(NSC681152) and Skp2-2^25^ (Fig. 4A), both of which have been reported as Skp2-Cks1 inhibitors in previous literature. Then antibody-based HTRF HTS assay was used to detect the compound's inhibition to Skp2-Cks1. The IC_50_ of Skp2-1 and Skp2-2 were 58.68 ± 1.62 μM and 27.81 ± 1.44 μM respectively (Fig. 4B,C). So, this method is useful for Skp2-Cks1 activity detection and inhibitor screening.

### Verification the targeting of Skp2-2 in cell

**Figure 5:**
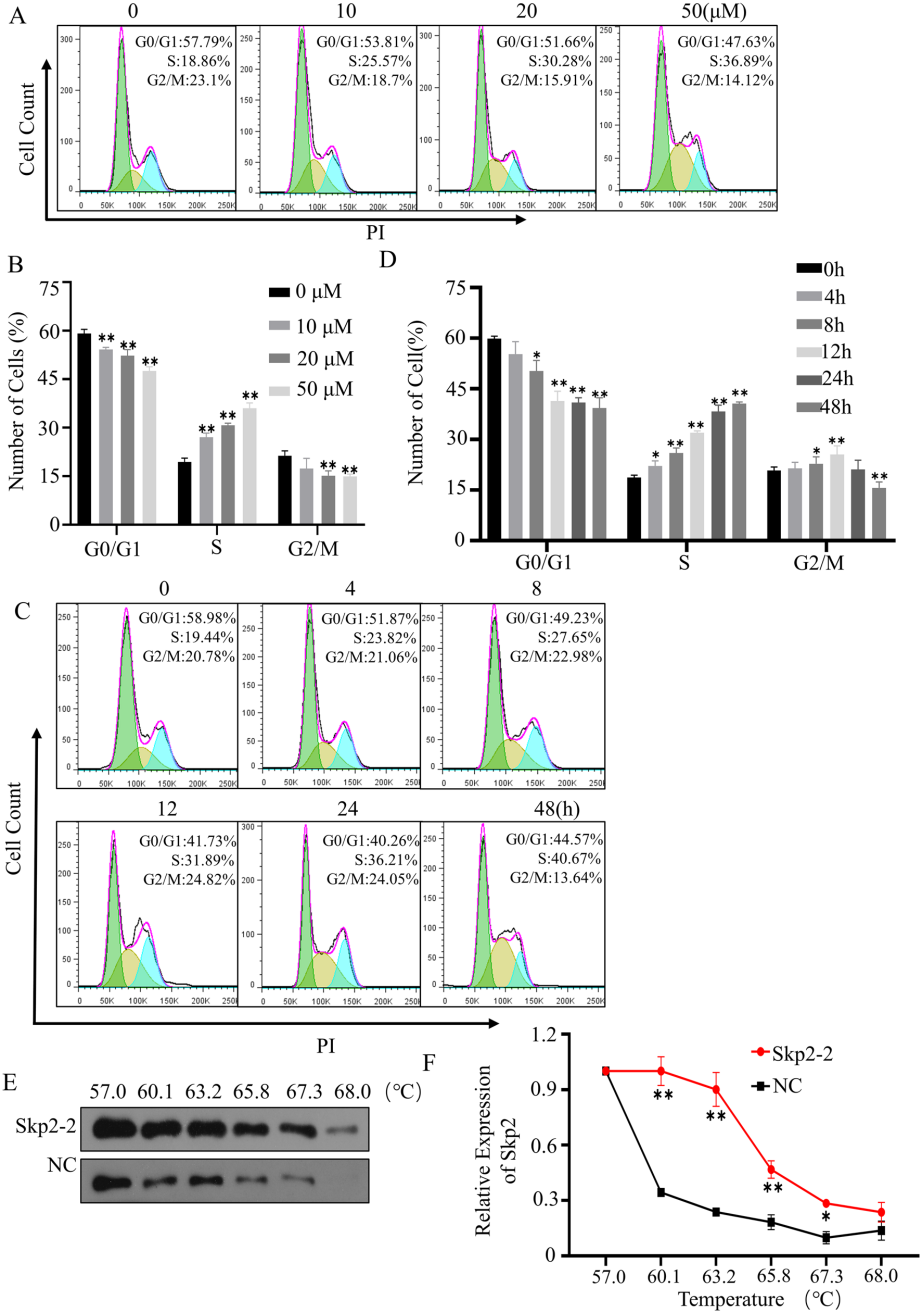
Skp2-2 inhibits cell cycle progression in SGC-7901. (**A**,**B**) SGC-7901 cells treated with increasing concentrations of Skp2-2 for 48 h. (**C**) (**D**) SGC-7901 cells treated with 50 μM Skp2-2 for 0–48 h. Fixed cells were collected and the cell cycle was measured by flow cytometry. (**E**,**F**) SGC-7901 cells treated with 50 μM Skp2-2 for 4 h, the stability of protein Skp2 was measured at different temperatures. The representative data of at least three independent experiments are shown, *P < 0.05, **P < 0.01. compared with the control group (mean ± SD, n = 3).

The compounds that have been screened for high throughput sometimes occurred off-target effects. It’s necessary to further testify the specificity of compound on Skp2 at both cellular and protein levels. The SGC-7901, a gastric cancer cell with high Skp2 expression, was selected for the subsequent verification experiment. The IC_50_ of Skp2-2 for SGC-7901 was 57 μM detected by MTT assay (Fig. 4D). As shown in the figure, Skp2-2 could significantly and dose-dependently reduce the protein expression of Skp2 (Fig. 4E,F). The p21 and p27 are downstream proteins of Skp2 and are vital for proliferation, survival, and periodic distribution of tumor cells^26–28^. The protein levels of p21 and p27 were significantly increased in cells treated with the compound Skp2-2. When the compound Skp2-2 was 50 μM, the protein Skp2 expression level reduced to 10%, and the expression levels of p21 and p27 were increased by about 20 times and 10 times, respectively. Next, we examined the changes of various proteins in cells exposed to the compound Skp2-2 at different periods. As described in Fig. 4G, Skp2 expression decreased gradually and reached its lowest level at 48 h, only one-tenth of that of the untreated cells with the compound Skp2-2. p21 and p27 also increased in the same proportion (Fig. 4H). RT-PCR results revealed that Skp2-2 can also influence the mRNA levels of Skp2, p21, and p27 (Fig. 4I). As can be seen, the expression of Skp2 decreased significantly and dose-dependently at mRNA levels. Decreasing in Skp2 inhibited tumor cell growth and leads to cell S-phase arrest^29^. The cells treated with Skp2-2 showed significant S-phase arrest, with the proportion of S-phase increasing from 18.86% to 36.89% compared with the control group (Fig. 5A,B), and the proportion of decrease was positively correlated with concentration and time treated with the compound (Fig. 5C,D). As shown in Fig. 5E,F, the Skp2 is more stable in cells treated with the compound at a temperature range of 57 to 68 ℃. These results suggested that Skp2-2 is the targeted inhibitor for Skp2. So, this method is useful for inhibitor screening of Skp2-Cks1.

### Discovery and bioactivity evaluation of Skp2-Cks1 inhibitor SYK-031

**Figure 6:**
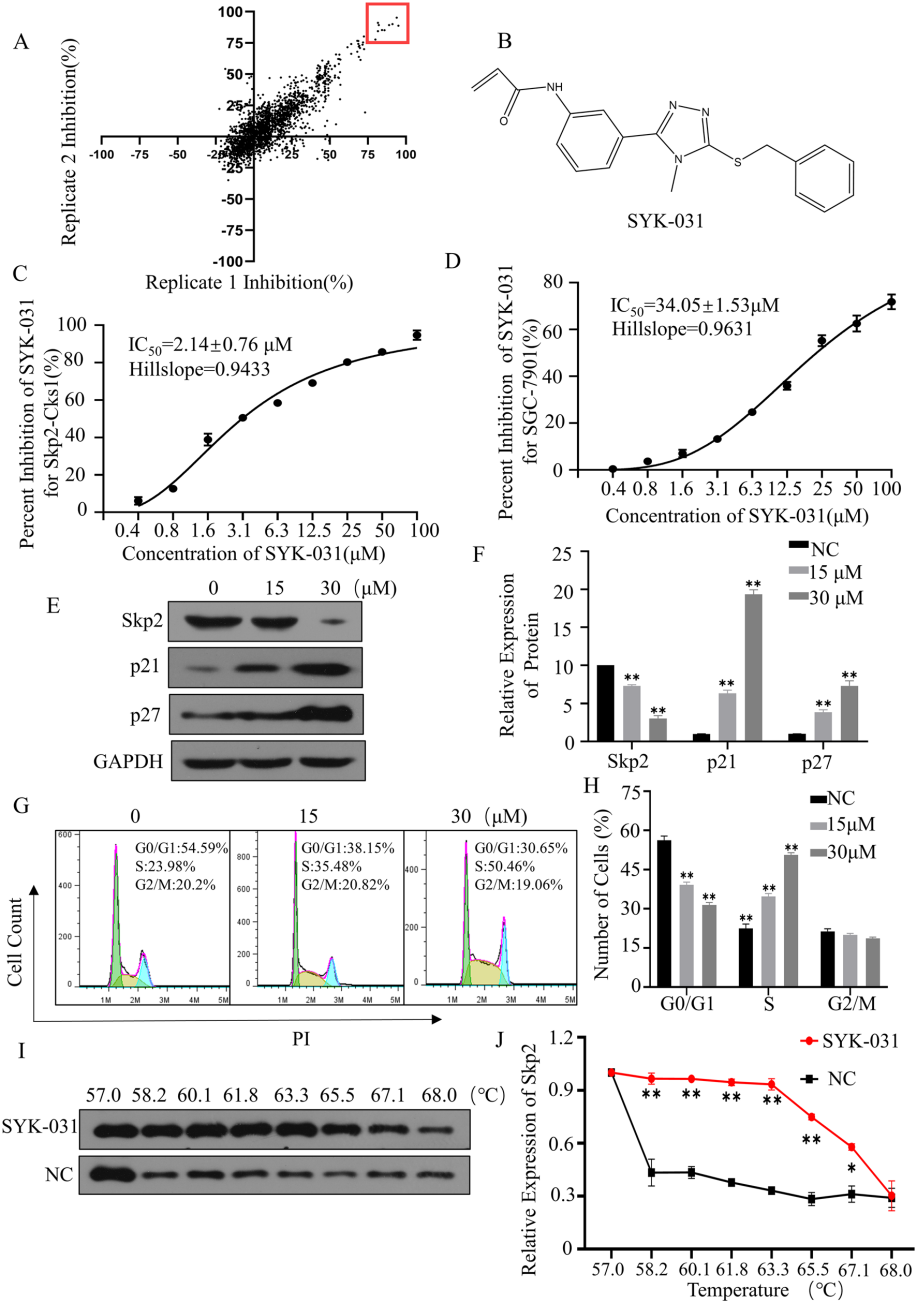
Discovery and bioactivity evaluation of compound SYK-031. (**A**) percent inhibition of 2118 compounds at 50 μM for Skp2-Cks1. (**B**) The chemical structure of compound SYK-031. (**C**) The percent inhibition of the compound SYK-031 at various concentrations in Skp2-Cks1. (**D**) The percent inhibition of the compound SYK-031 at various concentrations in SGC-7901. (**E**,**F**) SGC-7901 cells treated with increasing concentrations of SYK-031 for 48 h and the cells lysates were collected for Western blotting with the indicated antibodies. (**G**,**H**) SGC-7901 cells treated with increasing concentrations of SYK-031 for 48 h. Fixed cells were collected and the cell cycle was measured by flow cytometry. (**I**,**J**) SGC-7901 cells treated with 50 μM SYK-031 for 4 h, the stability of protein Skp2 was measured at different temperatures. The representative data of at least three independent experiments are shown, *P < 0.05, **P < 0.01. compared with the control group (mean ± SD, n = 3).

The lead compound SYK-031 was identified by preliminary screening of more than 2,000 compounds in our in-house diverse compound library using the Skp2-Cks1 high-throughput screening platform, and the compound SYK-031 showed excellent inhibitory activities against the binding of Skp2 and Cks1 (IC_50_ = 2.14 ± 0.76 μM) (Fig. 6A–C). The IC_50_ of SYK-031 for SGC-7901 was 34.05 μM detected by MTT assay (Fig. 6D). As shown in the figure, SYK-031 could significantly arrest the cell cycle at S phase and dose-dependently increase the protein expression of p21 and p27 in SGC-7901 cells (Fig. 6E–H). The cellular thermal sift assay showed the Skp2 is more stable in cells treated with the compound at a temperature range of 57 to 68 ℃, which indicated compound SYK-031 could specifically target Skp2 in cancer cells(Fig. 6I,J).

## Discussion

Skp2, as a short-lived protein, is regulated by UPS during cell life and degraded by the ubiquitin–proteasome pathway. Because most of its substrates are associated with tumor inhibition, Skp2 is considered to be a cancer-related protein, and its high expression in most cancers also reveals that^30^. Skp2 plays an important role in different cancers due to its significant regulation of cell cycle progression and its direct and indirect influence on the initiation and development of tumors through the activation of multiple signaling pathways and transcription factors, such as the PI3K/AKT pathway, IL-6/JAK2/STAT3 pathway, SP1, FOXM1, etc^31^. The p27 is a cell cycle regulator and can lower the kinase activity of Cdk2 (cyclin-dependent kinase 2) by binging with the Cdk2/cyclin A complex, and causing cell block from G1 phase to S phase^32,33^. Previous studies revealed that Skp2 was upregulated in the G2 phase and involved in the degradation of p27^34^. Cyclin‐dependent kinase subunit 1(Cks1) was a small cohesive protein and can greatly enhance the binding affinity of p27 to Skp2^18^. When phosphorylated at threonine on p27 protein 188 sites, Skp2 interacts with Cks1 and leads to p27 degradation through the ubiquitin–proteasome system which promotes tumor proliferation and migration^35^. It was reported that Skp2 is highly expressed at protein and mRNA levels in a variety of cancer tissues and mediates tumorigenesis. And this was consistent with the results that downregulation of p27 predicted poor prognosis of tumors^36,37^. The development of Skp2-Cks1 inhibitors might provide a novel and potential target for Skp2-overexpressed tumor patients^38^.

Skp2 has attracted increasing attention as an ideal antitumor drug target, and some small molecule inhibitors of Skp2 have been reported in the literature, such as SMIP004(Skp2)^39^, 6-O-angeloylplenolin (Skp1-Skp2)^40^, C1, C20(Skp2-p27)^41^, M1(Skp2-p300)^42^, NSC681152 (Skp2-Cks1), Compound 22d(Skp2-Cks1)^25^, etc. Most of these small molecule inhibitors act on the Skp1/Skp2-Cks1-p27 axis, thereby triggering intracellular signaling and inhibiting the growth and proliferation of tumor cells. The high-throughput screening platform for most Skp2 inhibitors was found to screen compounds by detecting changes in the fluorescence signal of a single protein after drug action. Although this method can directly observe the influence of intracellular protein expression of the drug, it’s susceptible to other factors and lead to false-positive results.

HTRF technology-based Skp2-Cks1 high-throughput drug screening platform has been previously reported, but the detection reagents used are different from ours. At the same time, we also carried out some optimization and improvement based on the previous researches. In the previous literature, the reagents used were Eu with FLAG label and APC with GST label. APC (phycocyanin, XL665) is a large hetero hexameric edifice of 105 kDa, cross-linked after isolation for better stability and preservation of its photophysical properties in HTRF assays. But in this study, the recombinant proteins we used were 71 kDa and 10 kDa, respectively. The larger molecular weight of XL665 may cause a certain steric influence on the binding of the target protein, thus affecting the protein binding and HTRF value. The reagents we used were GST-Eu and His_6_-d2. The d2 is the second-generation receptor optimized based on XL665. Based on inheriting a series of photophysical properties of XL665, the molecular weight of d2 is only about 1 kDa, which can also solve the possible influence of steric hindrance. After the optimization of reagents and reaction conditions, when the concentrations of GST-Skp2 and His_6_-Cks1 were 20 nM and 30 nM respectively, the HTRF value could reach the optimal level. However, in the previous literature, the concentrations of GST-Skp2 and FLAG-Cks1 were 140 nM and 160 nM respectively, which may be due to reduce the effect of steric hindrance and made the protein binding more fully. On the other hand, we optimized the purification method of the target protein, which greatly increased the purity and activity of the target recombinant protein.

When screening of Skp2-Cks1 target inhibitors, there were mainly two methods: AlphaScreen^43^ assay and HTRF^17^ assay, both were made up of donor beads, receptor beads, and the molecular complex of interaction. They can stimulate the donor beads by excitation light of specific wavelengths, thereby causing the energy cascade transfer reaction and the receptor beads emitting energy. Compared with the HTRF assay, the AlphaScreen assay had a relatively high window range because a donor bead can release more than 6,000 singlet oxygen molecules, and the AlphaScreen assay had a dynamic range of over 200 In the previous literature about the AlphaScreen system of Skp2-Cks1. AlphaScreen technology has its advantages, but there are limitations. The capture of monomer oxygen molecules by some compounds reduces the optical signal, and the photobleaching effect of donor beads makes it impossible to detect multiple times. The HTRF technology could greatly solve the deficiency of AlphaScreen Assay. Due to the long half-life of lanthanides, HTRF technology has a low background and can effectively avoid the interference of non-specific fluorescence and false-positive results. However, the disadvantage of HTRF is the small window range, which makes the sensitivity too low in the drug screening process. Combining the two screening methods to screen Skp2-Cks1 target inhibitors may be a good solution and promote the development of Skp2 small-molecule inhibitors.

We selected an inhibitor of Skp2 that has been reported to verify the accuracy of this screening platform, and the protein level and mRNA level results showed that the compound of Skp2-2 is the targeted inhibitor for Skp2. There are not currently Skp2-Cks1 inhibitors for the treatment of malignant tumors in clinic. The established High-throughput screening HTRF assay of Skp2-Cks1 was used for screening of compound libraries. After screening, we obtained the lead compound from 2000 compounds, and its IC_50_ was about 2.14 μM at the enzyme activity level, but it did not show a good effect in cells. Therefore, in the follow-up work, we will continue to optimize its structure to get better small molecule inhibitors.

In summary, we have built a small molecule inhibitor screening method based on HTRF technology, which can screen the target inhibitors of Skp2-Cks1. We will continue to optimize based on the screened lead compounds to find promising small molecule inhibitors of Skp2-Cks1 for clinical application. At the same time, based on analyzing different screening technologies, we try to combine HTRF with AlphaScreen, to make the high-throughput screening system for small molecule inhibitors of Skp2-Cks1 more efficient and convenient.

## Supplementary Information

Supplementary Information.
