# Publisher Correction: Establishment of high-throughput screening HTRF assay for identification small molecule inhibitors of Skp2-Cks1

**DOI:** 10.1038/s41598-021-01776-4

**Published:** 2021-11-10

**Authors:** Kaizhao Hu, Xiao-Jing Li, Moges Dessale Asmamaw, Xiao-Jing Shi, Hong-Min Liu

**Affiliations:** 1 State Key Laboratory of Esophageal Cancer Prevention and Treatment, Key Laboratory of Advanced Drug Preparation Technologies, Henan Key Laboratory of Drug Quality Control and Evaluation, Ministry of Education of China, School of Pharmaceutical Sciences, Zhengzhou University, Zhengzhou, 450001 Henan China; 2 Laboratory Animal Center, Academy of Medical Science, Zhengzhou University, Zhengzhou, 450052 Henan Province China

Correction to: *Scientific Reports* 10.1038/s41598-021-00646-3, published 26 October 2021

The original version of this Article contained an error in the spelling of the author Xiao-Jing Shi which was incorrectly given as Xiaa-Jing Shi.

The original Article has been corrected.

