# Supplementary material for: Establishment of high-throughput screening HTRF assay for identification small molecule inhibitors of Skp2-Cks1
Source: Sci Rep. 2021 Oct 26;11:21105. doi: 10.1038/s41598-021-00646-3 (PMC8548536; doi:10.1038/s41598-021-00646-3)

1.Western blot original image for figure 4E

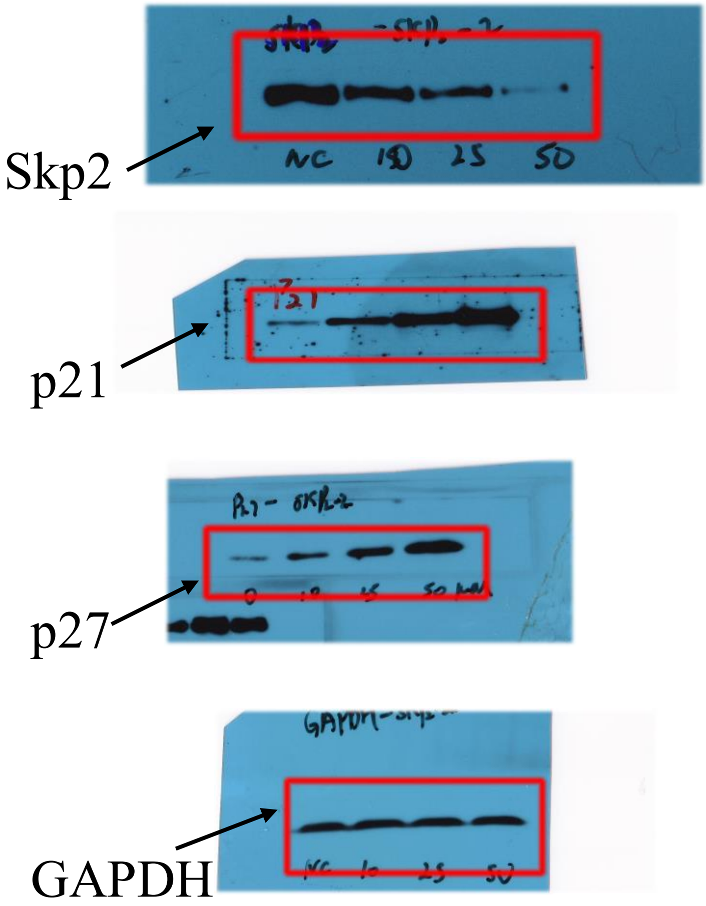

2.Western blot original image for figure 4G

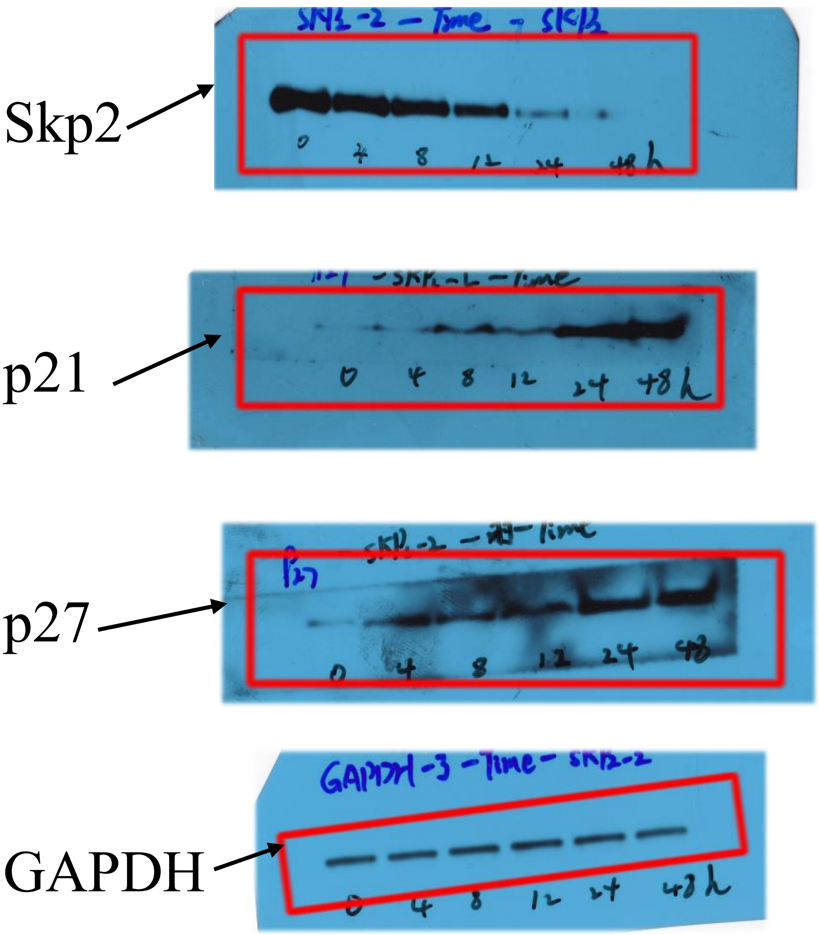

3.Western blot original image for figure 5E

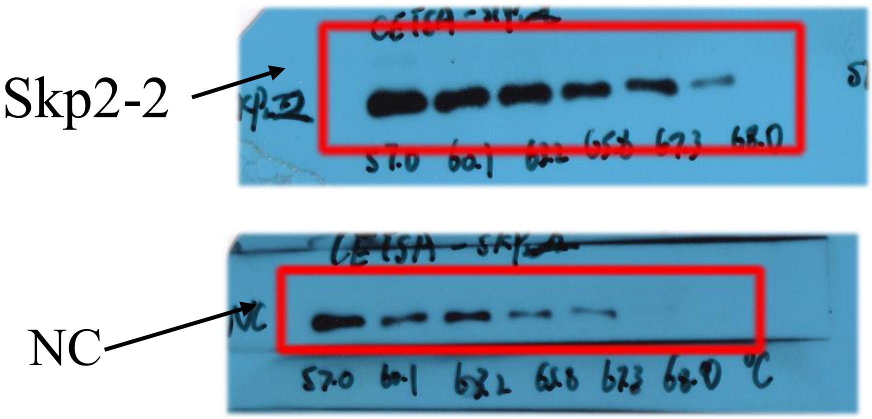

4.Western blot original image for figure 6E

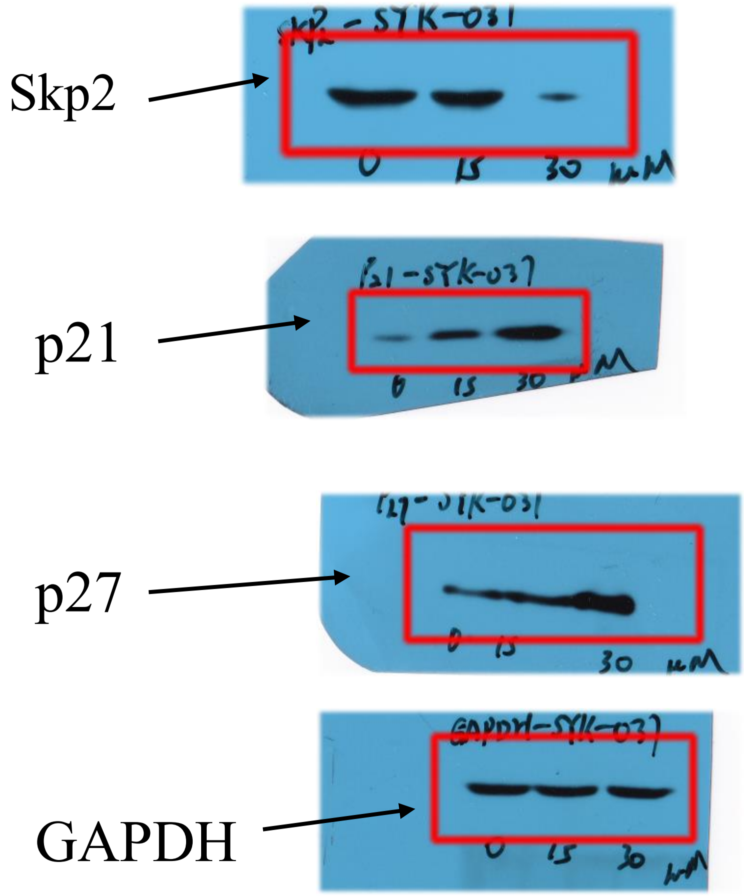

5.Western blot original image for figure 6I

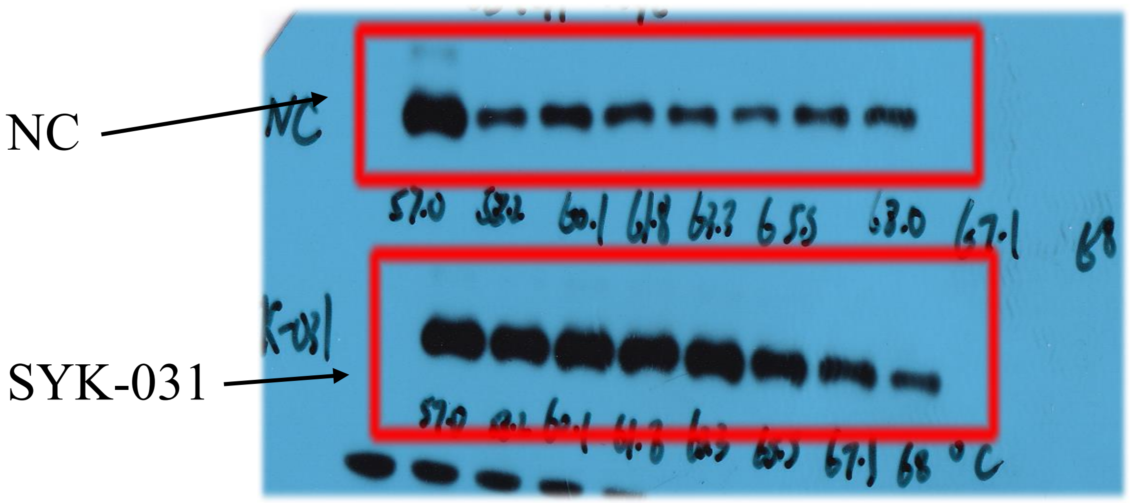

Supplement: Supplementary file 1 — Supplementary Information. [file 41598_2021_646_MOESM1_ESM.pdf]
